# Ethnic studies increases longer-run academic engagement and attainment

**DOI:** 10.1073/pnas.2026386118

**Published:** 2021-09-07

**Authors:** Sade Bonilla, Thomas S. Dee, Emily K. Penner

**Affiliations:** ^a^College of Education, Center for Student Success Research, University of Massachusetts, Amherst, MA 01003;; ^b^Graduate School of Education, Stanford University, Stanford, CA 94305;; ^c^Research Associate, National Bureau of Economic Research, Cambridge, MA 02138;; ^d^School of Education, University of California, Irvine, CA 92697

**Keywords:** ethnic studies, anti-racist education, high school graduation

## Abstract

Increased interest in anti-racist education has motivated the rapidly growing but politically contentious adoption of ethnic studies (ES) courses in US public schools. A long-standing rationale for ES courses is that their emphasis on culturally relevant and critically engaged content (e.g., social justice, anti-racism, stereotypes, contemporary social movements) has potent effects on student engagement and outcomes. However, the quantitative evidence supporting this claim is limited. In this preregistered regression-discontinuity study, we examine the longer-run impact of a grade 9 ES course offered in the San Francisco Unified School District. Our key confirmatory finding is that assignment to this course significantly increased the probability of high school graduation among students near the grade 8 2.0 grade point average (GPA) threshold used for assigning students to the course. Our exploratory analyses also indicate that assignment increased measures of engagement throughout high school (e.g., attendance) as well as the probability of postsecondary matriculation.

## Significance

Anti-racist curricula and teaching methods are a potentially potent way for schools to better promote a just society and improve educational outcomes for low-income students and students of color. Ethnic studies (ES) courses in K–12 schools are an increasingly common and prominent example of such culturally relevant and critically engaged practice. Proponents tout the benefits of ES for increasing student engagement and academic outcomes, yet there is little causal evidence supporting these claims. In this study, we use a preregistered regression-discontinuity research design to identify the longer-term impacts on educational attainment and engagement of being assigned to an ES course in grade 9. We find that assignment to ES substantially increased high school graduation, attendance, and the probability of enrolling in college.

Recent, high-profile incidents of police violence in the United States have brought increased public attention to contemporary manifestations of systemic racism in US society and catalyzed a corresponding interest in how key institutions such as schools can better promote a just society. In education, this focus has included renewed attention to the design and impact of curricula and teaching methods with distinct anti-racist features (1). A prominent example is the growing interest in ethnic studies (ES) courses. In general, ES courses focus on the histories of marginalized communities, the promotion of students’ critical awareness of social issues, and the encouragement of civic engagement and community-responsive social justice. Today, a growing number of school districts and states are offering ES courses to K through 12 students and, in some cases, passing ES course requirements for all students (2, 3).

The increased interest in ES courses has engendered controversy. Critics argue that ES courses are a biased and politically charged indoctrination that constitutes a form of “reverse racism” (4, 5). Proponents counter that the need for ES courses needs to be understood in the historical context of the US, which has consistently included the overt oppression and marginalization of racialized and ethnic minorities (6). Over 50 y ago, the ES discipline emerged in response to this historical awareness and the recognition that the perspectives of communities of color had been excluded from the nation’s curricula. During and after the social movements of the 1960s, communities of color and student coalitions like the Third World Liberation Front advocated for ES as a way to increase educational access, inclusion, and autonomy for faculty and students of color and to field new curricula that reflected the experiences and struggles of historically marginalized groups in the United States (6, 7).

The current debate over ES courses also turns on opposing claims about their educational effects. Critics argue that ES courses lack academic rigor and replace “reasoning through logic and consideration of evidence” with “a vague deconstruction of power relationships” (4, 8). In contrast, ES proponents often make strong claims about how ES courses can substantially improve academic engagement, critical thinking, and longer-run student success (6, 9). Numerous qualitative studies have presented evidence that ES courses have positive effects on student engagement (i.e., sense of identity, school belongingness), student achievement, and high school graduation (see ref. 6 for a summary). However, there are only two quantitative studies examining the relationship between taking an ES course and student outcomes. Cabrera, Milem, Jaquette, and Marx (10) found that voluntary participation in Tucson’s Mexican American Studies (MAS) program was associated with increases in standardized test pass rates and high school graduation.

This promising descriptive evidence is further corroborated by Dee and Penner’s (11) quasi-experimental evaluation of the effects of the San Francisco Unified School District’s (SFUSD) pilot grade 9 ES course. Using a regression-discontinuity (RD) design, they found that participation in the ES course substantially increased attendance, credits earned, and course performance during grade 9. That study relied on comparisons across students whose grade 8 grade point average (GPA) placed them below or above the 2.0 threshold used for course assignment during the developmentally sensitive transition to high school. However, the fade out of short-term gains is common in educational interventions (12), so these effects may not be sustained when students return to conventional academic programs. Alternatively, if students subsequently encounter supportive learning environments that continue to foster personal and academic growth, participation in the ES course may lead to a reinforcing cycle of improved engagement and academic success that sustains or enhances longer-run educational outcomes (13).

In this study, we present results from a preregistered RD design (14) that examines the longer-run effects of the ES course among the students in the original Dee and Penner (11) sample.* We find evidence that ES enrollment substantially increased the probability of graduating from high school, an outcome with important life-long benefits including health and economic success. We also find complementary evidence that the grade 9 ES course promoted student engagement and persistence throughout high school (i.e., enrollment, attendance, and credits earned) and that these gains extended to postsecondary enrollment. Finally, we note some important caveats of our research sample and design that have implications for our study’s generalizability and efforts to replicate and scale these results. First, our study relies on data from five cohorts that opted to offer ES in a pilot program where teachers received intensive training in developing ES curricula. Second, participating schools served lower-performing students who were more likely to have disabilities and identify as Latinx than the general SFUSD population. Third, although this smaller-scale implementation of ES has strong causal warrant due to the RD design, the impacts are defined for students near the 2.0 GPA threshold who complied with their ES assignment.

## Background

In 2010, SFUSD’s Board of Education unanimously passed a resolution, requesting that the Superintendent fund and implement a pilot ES course in SFUSD high schools. The resolution also asked that this course meet the University of California’s certification as a college-preparatory elective. A “collective” of 10 social studies teachers developed and tested course materials for a year-long grade 9 course with support from faculty at the San Francisco State University and the District’s Office of Learning Support and Equity (15). Several teachers from this collective became the first to formally implement the course in several high schools between the 2010 to 2011 and 2013 to 2014 school years.

The units in this course focused on themes of social justice, anti-racism, stereotypes, and social movements led by people of color from US history spanning the late eighteenth century until the 1970s. The curriculum incorporated elements of histories and political struggles of multiple racial and ethnic groups, many of which are not traditionally represented in US social-studies content. For example, students in this course examined the genocide of Native Americans in California, community resistance in Chinese and Latinx neighborhoods in California, and labor organizing during the Great Depression and World War II among African Americans and Filipino Americans. The course also encouraged students to explore how social constructions of race, ethnicity, and culture shaped their individual identity, their family and community histories, and required students to design and implement participatory-action projects based on their study of racialized and ethnic relations in their local communities. The learning objectives of the course included student knowledge of and ability to combat racism and other forms of oppression, increased student commitment to social justice, and improvement of student pride in their own identities and communities. In addition to the civic and psychological goals of the ES program, the pilot’s stated intent was to close achievement gaps and reduce dropout rates (15, 16).

The conceptual foundations for ES courses are closely related to an influential literature in education research on “culturally relevant pedagogy” (CRP). The definition of CRP stresses three broad features of culturally relevant instruction (17). These include both prioritizing students’ academic success and providing “a way for students to maintain their cultural integrity” [pp. 476 (17)]. Third, CRP emphasizes cultivating students’ critical abilities “to recognize, understand, and critique current and social inequities” [pp. 476 (17)]. ES has strong parallels to CRP because it centers the experiences and narratives of people of color, honors their cultural assets, develops strong relationships between students and teachers, and provides students with tools to critique inequality (18). ES also places a unique emphasis on acting to eliminate systemic oppression (6, 9, 18, 19). In this way, ES harnesses CRP and is explicitly anti-racist.

Dee and Penner (11, 20) discuss how CRP and ES can be understood within other social-scientific traditions. For example, several social–psychological field experiments find positive academic effects of brief student-level interventions that shape identities in the schooling environment. These include forewarning students about stereotypes (21), affirming students’ personal values (22), promoting social belongingness in school (23), and emphasizing both the capacity to learn (24) and external attributions for their life challenges (25). ES resembles an unusually intensive psychological intervention because it includes elements of these approaches, targets them to students transitioning to a new school, and delivers them on a sustained year-long basis. Furthermore, because ES is a classroom-level initiative, it may promote the supportive environments that are important for the successful replication of student-level interventions (13).

Long-standing sociological (26, 27) and, more recently, economic (28) perspectives on social identity also provide related conceptual frameworks for understanding ES. In general, these literatures have stressed how social identity has important behavioral consequences. For example, the ground-breaking work of DuBois (27) introduced the term “double consciousness” to explain the defining and burdensome “strife” marginalized people can experience when they simultaneously understand their self-worth and the contrary perceptions of a racist environment. Motivated by this, we adapt a social-identity model (28), which postulates that experiencing school as a prejudiced environment can result in reduced academic engagement (11, 20). These frameworks also indicate how ES can attenuate this dynamic and increase the engagement of marginalized students by promoting belongingness, affirmation, and high expectations within otherwise alienating schooling environments.

The motivation and design of ES courses also has strong parallels with several conceptual frameworks from community psychology. For example, critical consciousness theory likewise underscores the importance of promoting social analysis so that youth develop awareness of how social injustice operates and use that understanding to advocate for structural political and economic reform (29). Similarly, empowerment theory (30) emphasizes self-determination through approaches that privilege concrete goal-oriented strategies for affecting social change. Lastly, theories of sociopolitical development extend these conceptualizations by positing that individuals must develop the appropriate knowledge, analytic and emotional capacity of both individual-specific and systemic-level oppression to engage in effective political activism (31, 32). These supporting theories highlight the theoretical basis for a well-designed and implemented ES course to seed transformational change.

An extensive empirical literature on ES has stressed its capacity to catalyze school engagement and learning among students who have historically experienced academic environments as hostile spaces (6, 9, 18). However, most of this evidentiary base relies on close qualitative or descriptive assessments of small numbers of teachers and schools (9, 33–39). Only two studies complement this literature with larger-scale quantitative evidence. Cabrera et al. (10) find that voluntary student participation in Tucson’s MAS program is positively correlated with increased high school graduation. Using an RD design, Dee and Penner (11) provide causal evidence on the academic effects of SFUSD’s grade 9 ES course. This quasi-experimental study leveraged a unique institutional rule: several high schools assigned grade 9 students to the ES course if they had a grade 8 GPA below 2.0. This causal study indicated that the encouraged students were more likely to take the ES course and had substantial improvements in important, short-term outcomes (i.e., attendance, GPA, and credits earned) at the end of ninth grade.

Though the existing evidence suggests ES courses have proximate positive effects on students, there is no causal evidence on longer-term effects. It is reasonable to suspect that the short-term gains of the grade 9 ES course may dissipate as students return to conventional academic programs and environments. In some contexts, researchers have observed the “fade out” of effective educational interventions on academic outcomes (40–42), while in others they persist into adulthood (43). Whether ES courses have longer-term effects is an open empirical question that depends on multiple factors. For example, ES courses may have sustained or even growing effects if they catalyze reinforcing processes of academic motivation, engagement, and success that are sustained by supportive learning environments (e.g., high-quality teachers, engaging curricula, supportive school environments). However, the longer-term impact of an ES course may also depend on whether the protection it affords students at a vulnerable time of transition and identity formation (e.g., ninth grade), gives them a sense of purpose, coping mechanisms, and tangible skills to navigate a new school environment.

## Methods and Materials

### Data.

We use the focal sample examined by Dee and Penner (11). It consists of five unique school year cohorts of grade 9 students in SFUSD from academic year (AY)2011 to 2012 through AY2013 to 2014 (*n* = 1,405). The research was approved by the Institutional Review Boards of Stanford University and the SFUSD. We utilized preexisting, deidentified student records and did not conduct an experiment. As such, we had no informed consent procedure. We focus on these cohorts because their schools used a discontinuous assignment rule, automatically assigning ninth graders to the ES course if their grade 8 GPA was below 2.0.^†^ This assignment rule forms the basis for the RD design we implement.

We acquired longitudinal data on students from three broad sources. First, SFUSD’s administrative files include a variety of baseline traits as well as data on students’ progression through high school. Second, to measure high school graduation, we complement SFUSD data with data from the California Longitudinal Pupil Achievement Data System (CALPADS). And third, we measure college matriculation using student-level data from the National Student Clearinghouse (NSC).

The baseline data include binary indicators for sex and whether the student was Black, Latinx, Asian, or White. Additional baseline (i.e., grade 8) covariates include their attendance rate, indicators for special education status, English learner status, and having been suspended. These covariates also include the assignment variable used to determine ES encouragement (i.e., grade 8 GPA excluding physical education), centered at 2.0. We create a binary indicator identifying the “intent to treat” (ITT): whether the student had a GPA below 2.0. SFUSD’s transcript data also allowed us to identify students who took the ES course in ninth grade.

Table 1 provides the summary statistics for our sample. The demographic traits of these students parallel the district’s distinctive diversity. Just over 60% of our sample identify as Asian compared to 5% of students nationally. Latinx students comprise 23.1% of our sample, similar to the 27% enrolled nationally in K through 12 schools. Black students in our sample, 6.3%, are underrepresented compared to 15% of US public school students (44). Female students comprise only 41.7% of our sample, due to their higher representation among students excluded for having a perfect 4.0 grade 8 GPA and their disproportionate enrollment in non-ES pilot, selective magnet schools. Because our study sample includes schools that opted into the ES pilot for the purpose of raising student achievement of academically lower-performing students, our sample includes students that, on average, had lower grade 8 test scores, were more likely to be Latinx, and to receive supports for documented disabilities.‡ A small portion, 8.3%, had a grade 8 GPA below 2.0 while 12.7% ultimately enrolled in the ES course.

**Table 1.** Summary statistics of sample

Sample	Full sample		
	Mean	SD	*N*
Enrolled in ES, year 1	0.127	0.334	1,405
I(Grade 8 GPA < 2.0)	0.083	0.275	1,405
High school persistence			
Enrolled in SFUSD, year 2	0.974	0.158	1,405
Enrolled in SFUSD, year 3	0.949	0.221	1,405
Enrolled in SFUSD, year 4	0.928	0.258	1,405
Attendance, year 2	94.694	9.489	1,369
Attendance, year 3	93.054	10.667	1,333
Attendance, year 4	91.322	12.730	1,304
Credits earned, year 2	114.775	20.980	1,369
Credits earned, year 3	174.196	27.751	1,333
Credits earned, year 4	231.703	31.546	1,304
Educational attainment			
High school graduate, year 5	0.900	0.301	1,405
High school graduate, imputed	0.911	0.285	1,405
Enrolled in postsecondary, year 5	0.695	0.460	1,405
Enrolled in postsecondary, year 6	0.681	0.466	1,405
Baseline demographics			
Female	0.417	0.493	1,405
Black	0.063	0.242	1,405
Hispanic	0.231	0.421	1,405
Asian	0.601	0.490	1,405
Grade 8 special education	0.123	0.329	1,405
Grade 8 English language learner	0.184	0.388	1,405
Grade 8 ever suspended	0.018	0.132	1,405
Grade 8 attendance	96.684	3.141	1,405

The full sample includes *n* = 1,405 ninth graders from five cohorts who attended three district high schools beginning in the 2011 to 2012, 2012 to 2013, and 2014 to 2015 school years. I(Grade 8 GPA < 2.0) indicates a grade 8 GPA less than 2.0. High school graduation includes all students with a state-confirmed graduation status from any California public school (i.e., high school graduate, year 5). Our preferred imputed graduation status (i.e., high school graduate, imputed) imputes graduation for students who left the district and did not subsequently enroll in a California public school (i.e., enrolled in a private school, out-of-state public school, or left to another country). We impute a positive graduation status for those who seamlessly enrolled in postsecondary or who at the time of leaving the school district earned enough credits to meet internal district benchmarks for “on-track” to graduate. The high school graduation measure was the single confirmatory outcome preregistered (14).

We examine several outcome variables, including one preregistered confirmatory measure: high school graduation. Because SFUSD administrative records do not consistently track the educational attainment of the students who left the district, our measure relies on CALPADS data that identify graduation (or drop out) from any public high school (e.g., public, charter) in California. Using the CALPADS data, the completion status for 76 students out of the original 1,405 is unrecorded.^§^ Our preferred approach characterizes students as high school graduates if they met district-defined credit and course benchmarks for the district's high school graduation requirements. In *SI Appendix*, Table S1, we report versions of our main RD results using several different measures and find similar effects. The vast majority of the remaining 76 students, particularly those near the ITT threshold, are highly likely to be missing from the statewide data on high school completers because they dropped out of high school.^¶^ Studies based on similar administrative data (45) often identify such students as dropouts. We report results based on this approach in *SI Appendix*, Table S1.

However, it is possible that some of these 76 students actually graduated from a private or out-of-state high school. To examine the empirical relevance of this measurement concern, we also implement two more conservative approaches to identifying the educational attainment of students who do not appear in the statewide graduation files (or the NSC data). Under the first approach, we characterized as high school graduates any students who were on track for graduation at the time they were no longer observed in the data (*n* = 15). This on-track measure utilizes credit accumulation and specific course completion requirements (e.g., English 1) that students are expected to meet each semester of high school. For example, on-track students must accumulate at least 30 credits their first semester of high school and earn five credits in math, English, and physical education. SFUSD defines benchmarks for each semester of high school based on the district’s high school graduation requirements. This is the main measure of high school graduation used in our analysis. Under a second approach, we instead identified as high school graduates those students (*n* = 28) who were at least moderately on track for graduation at the time they no longer appeared in the administrative data (i.e., no more than one semester behind in progress toward graduation). In *SI Appendix*, Table S1, we report versions of our main RD results using all of these slightly different measures and find similar results.

We also examine several exploratory outcomes that capture high school persistence as well as postsecondary enrollment. Our persistence measures include binary indicators for enrollment in SFUSD 1, 2, and 3 y after the intervention year (i.e., enrolled in years 2 through 4). A student is defined as enrolled when they have transcript records and attendance records for at least one semester in a given school year. We also examine attendance rates and credits earned during each of these years, conditional on enrollment in SFUSD. These measures capture student persistence and are standard proxies for behavioral engagement in school (46, 47). The vast majority of students in our sample remained enrolled in the district in the first year after they entered ninth grade (i.e., 97.4%). Enrollment in the school district decreased by ∼2 to 3 percentage points in years 3 and 4, to 94.9 and 92.8%, respectively. Attendance also decreases throughout high school with the largest decrease occurring between the third and fourth year of high school (i.e., from 93 to 91%) but, on average, remains over 90% throughout high school. The last measure we examine is credits earned. Students are required to earn 220 credits to graduate. The average student in the school district earns over 230 credits by the end of their fourth year of high school, signaling that the majority of enrolled students meet the credit requirements for high school graduation.

Our final set of measures derive from postsecondary enrollment data from the NSC. These data are available for all students, regardless of district enrollment throughout the study period. Similar to national statistics, 69.5% of our sample enroll in postsecondary education (i.e., either a 2- or 4-y institution) immediately after high school (44). We also examine a second measure of postsecondary enrollment based on data a full year after on-time graduation (and 6 y after first participating in the ES course). On average, this enrollment measure decreases slightly to 68.1%.

### Methods.

We examine the longer-run effects of the ES course in an RD design based on SFUSD’s assignment rule to identify the rising ninth graders who were encouraged to take the course. The RD specification we estimate effectively compares the conditional outcomes of students with a grade 8 GPA just below 2.0 (i.e., ITT = 1) to students whose grade 8 GPA placed them just above this threshold (i.e., ITT = 0). Specifically, we estimate the parameters of the following equation:

$$Y_{ist}=\alpha +\beta I\left(G_{ist}<0\right)+f\left(G_{ist}\right)+\lambda X_{ist}+\eta_{st}+\varepsilon_{ist},$$

where $Y_{ist}$ is a student-level outcome (e.g., high school graduation) for student *i* in school *s* and in year *t.* The assignment variable, $G_{ist}$, is the grade 8 GPA centered at 2.0. We create a binary indicator for the ITT, $I\left(G_{ist}<0\right)$, that is equal to one for students whose GPA is less than 2.0 (and 0 otherwise). Thus, the parameter β represents the discontinuous change in outcomes when students are encouraged to take the ES course, conditional on $f\left(G_{ist}\right)$, a smooth function of the assignment variable. We also present results with controls for a vector of student-level baseline traits (i.e., $X_{ist}$) and fixed effects specific to each unique school year cohort (i.e., $\eta_{st}$). The term, $\varepsilon_{ist}$, is a mean-zero error term, and all our models rely on robust (i.e., Eicker–Huber–White) SEs.

Intuitively, an RD design like this identifies causal effects by leveraging the “as good as randomized” variation (48) in whether a student was just above or just below an arbitrary threshold that implies a sharp contrast in treatment. However, the causal warrant of such an RD design turns on several critical assumptions. In recent years, the available research guidance (49) has provided increasingly standardized recommendations for several types of ancillary evidence that critically examine these key assumptions. First, we note that, using these data, Dee and Penner (11) present both graphical and statistical evidence of a large and discontinuous change at the relevant threshold in the probability of enrolling in the grade 9 ES course. Specifically, RD estimates indicate that the probability of taking the course increases by a large and statistically significant amount (i.e., 27 percentage points) for students with grade 8 GPAs below the 2.0 threshold.^#^

A second key consideration is the “integrity” of the assignment variable in an RD design (49). The institutional knowledge of this process (e.g., the use of a predetermined variable for a ninth-grade assignment) suggests that the assignment variable could not be easily manipulated in ways that would confound the internal validity of the RD design. Consistent with this assumption, we find that a density test (50) does not reject the null hypothesis that the number of observations is smoothly distributed around the threshold.^||^ Third, because we observe our key outcome measure for the full sample, differential attrition is not an issue. Some of our exploratory outcome measures (i.e., attendance and credits earned) are only observed for students who remained enrolled. However, we present RD evidence that the probability of being enrolled in SFUSD is balanced around the ITT threshold for each of the years after entering ninth grade, which suggests that sample attrition is not differential.

An important, fourth source of evidence on the validity of an RD design concerns the balance of outcome-relevant, baseline covariates. If the variation in students’ position around the RD threshold is “as good as randomized,” we would expect that baseline student covariates do not exhibit discontinuities at the ITT threshold. Consistent with this, auxiliary RD regressions in which the baseline covariates used in this study are the dependent variables indicate that the covariate differences at the ITT threshold are small and statistically insignificant (*SI Appendix*, Table S2).** A closely related source of evidence is to examine graphically the relationship between the assignment variable and the outcomes. This graphical evidence provides both unrestrictive evidence on the possible effect of the ITT and an opportunity to assess whether any discontinuities are apparent at other thresholds that did not define a treatment contrast.

A fifth class of evidence on the validity of an RD design involves exploring the robustness of the results to alternative ways of modeling functional form (i.e., $f\left(G_{ist}\right)$). Our baseline specification conditions on linear splines of $G_{ist}$, allowing the assignment variable to have distinctive slopes above and below the ITT threshold. However, we also show our key results in specifications that add quadratic splines. Critically, we also show the results from nonparametric local linear regressions (LLR) using data from increasingly tight bandwidths around the ITT threshold. We also present our key results graphically with both the full sample of data as well as the subset of data in a tighter bandwidth (i.e., 1 SD of grade 8 GPA) around the ITT threshold. Furthermore, we show the results of specifications that use algorithmically chosen bandwidths (52, 53) and triangular kernel weights, which upweight observations that are closer to the ITT threshold (54).

We conclude with two important observations about the generalizability (i.e., “external validity”) of our estimates. First, a well-known and intuitive caveat about RD designs is that, because they rely on variation close to a specific threshold, the resulting estimates may not generalize to students whose baseline traits made them distant from this threshold. For example, because our research design leverages an assignment rule that encouraged academically at-risk students to take the ES course, our results do not necessarily speak to the impact of the ES course for students who had a high grade 8 GPA.

A second external-validity issue concerns the possible heterogeneity in the treatment effects we report. Our key RD estimates identify the reduced-form impact of the encouragement to take the ES course (i.e., ITT = 1). However, as often occurs in randomized experiments, we have partial compliance with this ITT. That is, in this “fuzzy” RD application, the treatment status of some individuals does not comply with their ITT. Some students (i.e., “always takers”) take the ES course even when their grade 8 GPA is at or above 2.0 (i.e., ITT = 0). And some students (i.e., “never takers”) do not take the ES course even when their grade 8 GPA is below 2.0 (i.e., ITT = 1). The “local average treatment effect” (LATE) theorem (55) states that, in the presence of partial compliance and treatment heterogeneity, approaches like ours identify causal effects specifically for the subgroup of “compliers” (i.e., those who take the ES course when ITT = 1 and do not when ITT = 0). As an intuitive example of why this heterogeneity might reasonably exist, suppose the type of student who would always take the ES course has positive but unobserved motivations and experiences that make the possible academic benefits of the ES course less relevant for them. Then, we would expect the academic benefits of the course to be larger for “compliers” than for “always takers.” We test for this heterogeneity by presenting results from a procedure recently introduced by Bertanha and Imbens (ref. 56; *SI Appendix*, Tables S9 and S10).

Lastly, there are two unique concerns related to this study setting that the effects measured may be attributed to other factors. As noted previously, the district utilized an early warning indicator (EWI) to identify students at risk of high school dropout and target them with additional academic supports, raising concerns that these results are indicative of effective EWI interventions and not ES eligibility. Dee and Penner (11) estimate auxiliary regressions at the EWI threshold in schools that did not pilot ES and found null effects indicating that EWI supports did not have meaningful impacts on student outcomes. We repeat this falsification exercise with our attainment and engagement outcomes and similarly conclude that there is no evidence that district EWI supports increased student outcomes.^††^ A secondary concern relates to the fact that only four teachers taught ES to sample cohorts. We may be concerned that the positive outcomes are due to particularly effective teachers. However, Dee and Penner (11) examined the outcomes of these teachers’ non-ES students using a teacher fixed-effect estimation strategy and found the teachers were not different from the regular distribution of district social studies teachers. We follow up on these results and estimate the longer-term effects by excluding the students of the one teacher who exhibited consistently large fixed effects (i.e., indicating above average effects on student outcomes), and we find that the positive effects for ES-eligible students remained.

## Results

### Enrollment, Attendance, and Credits.

We begin by presenting visual and parametric RD evidence on how assignment to the ES course influenced measures of high school persistence and engagement across each of the 3 subsequent years. Our study preregistration characterized this analysis as exploratory. Table 2 presents parametric estimates of the effect of ES eligibility on enrollment in SFUSD, as well as attendance and credits earned conditional on enrollment. We present these exploratory results both for the full analytic sample (i.e., *n* = 1,405) and the subset of the sample within one SD of the ITT threshold (i.e., within 0.67 eighth-grade GPA points of 2.0).

**Table 2.** Reduced-form RD effects on high school persistence, by year

*Dependent variable*	(1)	(2)	(3)	(4)	(5)	(6)
	Full sample			Bandwidth |Grade 8 GPA_i_| ≤ 0.67		
	Estimate	*n*	Mean	Estimate	*n*	Mean
Enrolled in district, year 2	−0.00987	1,405	0.974	0.0261	424	0.960
	(0.0419)			(0.050)		
Attendance, year 2	5.741*	1,369	94.7	9.672***	407	90.0
	(3.065)			(2.432)		
Credits earned, year 2	8.014*	1,369	114.8	16.62***	407	101.8
	(4.794)			(5.739)		
Enrolled in district, year 3	0.00203	1,405	0.949	0.0511	424	0.910
	(0.0516)			(0.060)		
Attendance, year 3	6.323**	1,333	93.1	9.677***	386	87.3
	(2.989)			(3.540)		
Credits earned, year 3	10.56	1,333	174.2	24.66***	386	156.1
	(6.570)			(7.913)		
Enrolled in district, year 4	0.0374	1,405	0.928	0.0822	424	0.868
	(0.0606)			(0.068)		
Attendance, year 4	7.158***	1,304	91.3	11.56***	368	84.7
	(1.799)			(3.728)		
Credits earned, year 4	15.29**	1,304	231.7	31.46***	368	212.7
	(7.724)			(9.067)		

Each cell contains the result of a separate regression of the effect of I(Grade 8 GPA_i_< 2.0) on high school persistence measures (i.e., district enrollment, attendance, and credits earned) from administrative data. All models include linear splines and a full set of demographic controls (i.e., student sex and race/ethnicity indicators) and grade 8 (i.e., prior to treatment status) special education identification, English learner status, attendance, and suspension history. Grade 8 GPA is centered at 2.0. Robust SEs are reported in parentheses. *P < 0.10, **P < 0.05, and ***P < 0.01.

**Fig. 1.**
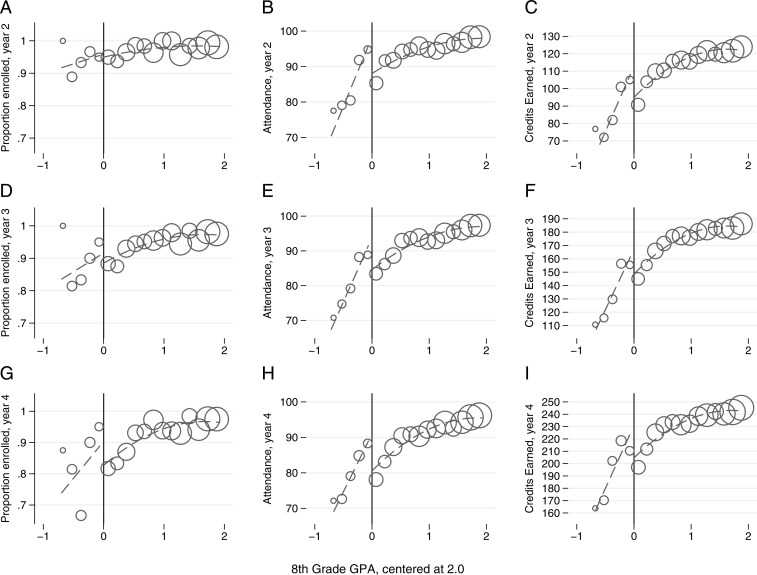
Measures of high school persistence. Graphs of the forcing variable (i.e., eighth-grade GPA) and measures of high school persistence, (*A*) enrolled in district in year 2, (*B*) attendance in year 2, (*C*) credits earned in year 2, (*D*) enrolled in district in year 3, (*E*) attendance in year 3, (*F*) credits earned in year 3, (*G*) enrolled in district in year 4, (*H*) attendance in year 4, and (*I*) credits earned in year 4. All graphs contain the full sample and use a bin width of 0.075 GPA points. For each graph, the circles represent the average outcome for students in a particular bin width of GPA points weighted by the number of student observations within each bin. The dashed line represents the fitted regression line with separate splines above and below the assignment threshold.

The results in Table 2 indicate that ES eligibility did not have statistically significant effects on subsequent SFUSD enrollment. However, these comparative results, though statistically imprecise, suggest that a discontinuity in enrollment did grow over time. By year 4, students with grade 8 GPAs below 2.0 were roughly 8 percentage points more likely to be enrolled than students to the right of the threshold (Table 2, column 4, *P* value = 0.24), which is supported by graphical evidence from Fig. 1 indicating that students assigned to take the ES course were more likely to remain enrolled over time.

In contrast, the results in Table 2 indicate that ES eligibility (i.e., ITT = 1) generated large and statistically significant increases in student attendance in all three posttreatment years. The estimated increases based on the full sample (i.e., 6 to 7 percentage points) are similar to the grade 9 effects reported by Dee and Penner (11). The estimated increases based only on students within a 1 SD bandwidth are somewhat larger (i.e., 10 to 12 percentage points). These results, which are also supported by a discontinuous increase in student attendance at the ES eligibility threshold presented in Fig. 1, suggest that the ES course led to sustained, and possibly growing, improvements in students’ behavioral engagement throughout high school.

However, successful progression through high school also depends on the successful accumulation of course credits. A semester-long course in the district typically confers five credits, and students commonly enroll in six to seven courses (i.e., 30 to 35 credits) per semester. During this period, SFUSD employed a 220-credit minimum in order to graduate. In the first-year study, ES-encouraged participants earned just over six additional credits than their ineligible peers [pp. 146 (11)]. Our full-sample results in Table 2 suggest that the credit gains among students with ITT = 1 were sustained and possibly grew over the next 3 y (i.e., 8 to 15 credits). These estimated gains are noticeably larger in the local linear regressions based on the 1 SD sample proximate to the threshold (i.e., 17 to 31 credits) and are supported by the graphical evidence in Fig. 1. These graphs also illustrate how these gains are salient to students’ likelihood of graduating from high school. In their fourth year, students on the ITT = 0 side of the threshold (Fig. 1*I*) had on average only 200 credits (i.e., roughly 20 credits short of the amount required for graduation). However, students just to the left of the threshold (i.e., those encouraged to take the ES course) were, on average, substantially closer to the number of credits required for high school graduation.

### Educational Attainment.

**Fig. 2.**
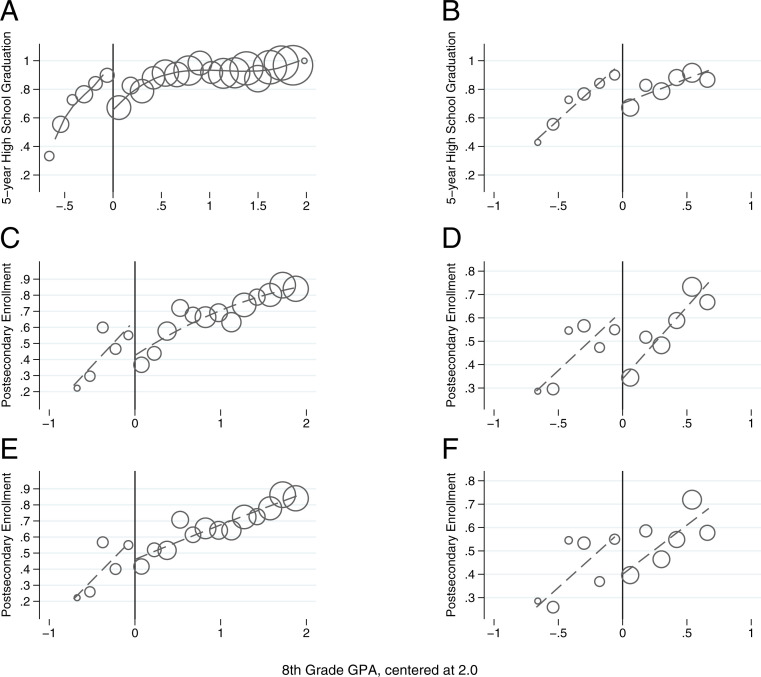
Educational attainment. Graphs of the forcing variable (i.e., eighth-grade GPA) and measures of educational attainment (i.e., high school graduation and postsecondary enrollment). (*A*) High school graduation utilizing full sample. (*B*) High school graduation, bandwidth ±1 SD. (*C*) Postsecondary enrollment year 5 utilizing full sample. (*D*) Postsecondary enrollment year 5, bandwidth ±1 SD. (*E*) Postsecondary enrollment year 6 utilizing full sample. (*F*) Postsecondary enrollment year 6, bandwidth ±1 SD. Graphs *A* and *B* utilize a bin width of 0.06. Graphs *C*–*F* use a bin width of 0.075. For each graph, the circles represent the average outcome for students in a particular bin width of GPA points weighted by the number of student observations within each bin. The dashed line represents the fitted regression line with separate splines above and below the assignment threshold.

Our main confirmatory analysis examines the effect of the encouragement to take the ES course on high school graduation. We report the key parametric results based on the full sample in the top of Table 3. These results indicate that ES eligibility generated substantial gains in high school graduation rates (i.e., from 16 to 19 percentage points). The graphical evidence in Fig. 2 supports these estimates and shows a substantial discontinuous jump in high school graduation at the ES-eligibility threshold. Similar to our measures of high school persistence and engagement, estimates based on a narrower bandwidth within 1 SD of the threshold are larger (i.e., 25 percentage points) than in the full sample.

**Table 3.** Reduced-form RD estimates of educational attainment

	(1)	(2)	(3)
Dependent variable:	*High school graduate*		
I(Grade 8 GPA < 2.0)	0.185**	0.175**	0.157**
	(0.073)	(0.073)	(0.073)
N	1,405	1,405	1,405
R^2^	0.101	0.107	0.151
Akaike's information criterion	323.5	321.8	259.5
Dependent variable:	*Postsecondary enrollment, year 5*		
I(Grade 8 GPA < 2.0)	0.163	0.162	0.134
	(0.101)	(0.0988)	(0.0920)
N	1,405	1,405	1,405
R^2^	0.0906	0.110	0.146
Akaike's information criterion	1,689.3	1,667.0	1,616.9
Dependent variable:	*Postsecondary enrollment, year 6*		
I(Grade 8 GPA < 2.0)	0.128	0.127	0.0976
	(0.100)	(0.098)	(0.092)
N	1,405	1,405	1,405
R^2^	0.0957	0.115	0.155
Akaike's information criterion	1,716.5	1,694.4	1,637.9
Demographic controls	—	Yes	Yes
Full controls	—	—	Yes

Each cell contains the results of a separate regression of the effect of I(Grade 8 GPA_i_ < 2.0) on graduation outcomes confirmed by the California Department of Education from any public school in the state. Our preferred measure of high school graduation includes imputed outcomes for district and state leavers (i.e., enrolled in private school, public school out-of-state or left country) who enrolled seamlessly in postsecondary or were on track to graduate based on internal district benchmarks for credits earned. All models contain linear splines. Demographic controls include student sex and race/ethnicity. The full controls include grade 8 (i.e., prior to treatment status) special education identification, English learner status, attendance and suspension history. Grade 8 GPA is centered at 2.0. Robust SEs are reported in parentheses. *P < 0.10, **P < 0.05, and ***P < 0.01.

These gains in high school graduation, however, may not translate to postsecondary enrollment given that ES eligibility targeted students with lower achievement in middle school. In the middle and bottom of Table 3, we present parametric estimates for postsecondary enrollment in years 5 and 6. These exploratory results suggest that the encouragement to take the ES course led to a large increase in college enrollment (i.e., 10 to 16 percentage points). However, in results based on the full sample, these point estimates are not statistically significant at conventional levels (Table 3, column 3, *P* = 0.16). In Fig. 2, we provide graphical evidence of the relationship between postsecondary enrollment and ES eligibility.^‡‡^ These graphs provide suggestive evidence of increased postsecondary enrollment at the ES threshold. Notably, our LLR estimates in *SI Appendix*, Table S1 (e.g., utilizing observations within 1 SD of the threshold) also indicate that there was a positive and statistically significant increase in postsecondary enrollment (i.e., 25 to 26 percentage points) at the ITT threshold.

In general, these results indicate that ES course-induced gains in high school graduation were large and possibly extended to increased postsecondary enrollment. We explore the robustness of these key findings in several alternative specifications. A uniquely salient concern is whether our RD results reflect the misspecification of the functional form that relates grade 8 GPA to these longer-run outcomes. The visual results in Fig. 2 provide unrestrictive evidence that suggests this is not a concern. However, we also complement this with the results of several different approaches to modeling $f\left(G_{ist}\right)$.

Table 4 presents the key results based on these specifications and for each measure of educational attainment. The first row of Table 4 presents the baseline results from Table 3 followed by estimates with quadratic polynomials and increasingly restrictive bandwidths. With respect to our core confirmatory inference about high school graduation, we see that the main results in Table 3 are quite robust to a range of bandwidth restrictions and estimation choices (e.g., weighting with triangular kernels).^§§^ In fact, when using data from tighter bandwidths around the threshold, the estimated effects of the ITT on high school graduation are often noticeably larger (i.e., 23 to 30 percentage points). With respect to our exploratory results regarding college enrollment, the results in Table 4 consistently suggest large, positive effects. However, these results are not always statistically significant.

**Table 4.** Reduced-form RD estimates of educational attainment with bandwidth restrictions

	(1)	(2)	(3)	(4)
*Dependent variable:*	*High school graduate, year 5*	*Postsecondary enrollment, year 5*	*Postsecondary enrollment, year 6*	
Sample	Estimate	Estimate	Estimate	*n*
Full sample, linear splines	0.157**	0.134	0.149	1,405
	(0.073)	(0.0920)	(0.091)	
Full sample, linear and quadratic	0.140	0.0618	0.202	1,405
	(0.122)	(0.253)	(0.253)	
|Grade 8 GPA_i_| ≤ 1.00	0.226***	0.192*	0.187*	634
	(0.079)	(0.098)	(0.098)	
|Grade 8 GPA_i_| ≤ 0.67	0.251***	0.260**	0.249**	424
	(0.087)	(0.105)	(0.104)	
|Grade 8 GPA_i_| ≤ 0.50	0.298***	0.268**	0.261**	341
	(0.096)	(0.115)	(0.115)	
|Grade 8 GPA_i_| ≤ 0.33	0.232**	0.107	0.117	208
	(0.110)	(0.150)	(0.153)	
|Grade 8 GPA_i_| ≤ 0.25	0.237*	0.259	0.316*	157
	(0.140)	(0.188)	(0.185)	
Kernel weights	0.256***	0.224**	0.235**	424
	(0.088)	(0.113)	(0.111)	
CCT optimal	0.162	0.185	0.370	126, 105, 106
	(0.161)	(0.232)	(0.225)	
IK optimal	0.256***	0.200	0.218*	357, 443, 438
	(0.092)	(0.122)	(0.121)	
Demographic controls	Yes	Yes	Yes	
Full controls	Yes	Yes	Yes	

Each cell contains the results of a separate regression of the effect of I(Grade 8 GPA_i_ < 2.0) on educational attainment (i.e., high school graduation and postsecondary enrollment). High school graduation is our preferred measure that includes imputed outcomes for district and state leavers (i.e., enrolled in private school, public school outof-state or left country) who enrolled seamlessly in postsecondary or were on track to graduate based on internal district benchmarks for credits earned. CCT= Calonico, Cattaneo, and Titunik; IK =Imbens and Kalyanaraman. Kernel, CCT, and IK estimates utilize triangular kernels. All other estimates utilize uniform weights. Demographic controls include student sex and race/ethnicity. The full controls include grade 8 (i.e., prior to treatment status) special education identification, English learner status, attendance, and suspension history. Grade 8 GPA is centered at 2.0. Robust SEs are reported in parentheses. *P < 0.10, **P < 0.05, and ***P < 0.01.

We also explored the robustness of our key results to alternative definitions of high school graduation. Our preferred measure complements the state-confirmed data with data from the NSC and, for a small number of observations, an imputation based on their on-track status when they attritted from the data (i.e., row 3 in *SI Appendix*, Table S1). We show in *SI Appendix*, Table S1 that our key results are quite similar when using alternative definitions of high school graduation (e.g., only using state and NSC data).

Finally, we assess whether any discontinuities in outcomes are apparent at other thresholds that did not define a treatment contrast and find no evidence of effects when conducting “placebo tests.” We conduct these robustness checks with two distinct samples. First, we use the main study sample and estimate the effect at GPA thresholds above and below 2.0 (i.e., placebo thresholds). Second, we utilize a sample of SFUSD students at high schools that did not offer ES courses and estimate the effect of being eligible for district EWI supports (e.g., tutoring) for lower-performing students and find no evidence of impacts (*SI Appendix*, Tables S4 and S5). Lastly, we follow Grembi and coauthors (57) and estimate a “difference in discontinuity” design where we combine the ES pilot and the non-ES school samples and estimate an RD model that differences out the corresponding threshold effects of the non-ES pilot school sample (*SI Appendix*, Table S6). These three supplementary analyses support the conclusion that encouragement to enroll in ES is responsible for the documented positive effects in increased high school engagement and educational attainment.

### Heterogenous Treatment Effects.

The original study found that there were consistently positive effects across student racial and ethnic groups and for both males and females. While the results indicated that ES eligibility was not harmful to any one demographic group, Dee and Penner (11) found that some students, particularly males and Latinx students experienced larger positive effects compared to female and other racial and ethnic groups. In *SI Appendix*, Table S7, we explore treatment heterogeneity using our high school persistence and engagement measures, and in *SI Appendix*, Table S8 we examine the effects on educational attainment.

Our subgroup specific effects on high school graduation indicate that all groups of students experienced gains. Both ES-eligible male and female students experienced positive and statistically significant gains in high school graduation, 15 and 23 percentage points, respectively (*SI Appendix*, Table S8). Asian and Latinx students demonstrated gains in high school graduation (i.e., 19 and 11 percentage points) though the estimates for Latinx students are statistically imprecise due to the lower representation of those students in our sample (i.e., 23% or 324 observations). The scale of this multischool pilot results in a relatively small sample size, and this limits our ability to examine heterogeneity in treatment effects for some racial/ethnic groups (i.e., whites and Blacks).

Another policy-relevant form of treatment heterogeneity is implied by the LATE Theorem (55). That is, in applications like ours where there is only partial compliance with the ITT, the resulting causal estimate may only be relevant for the subpopulation of “compliers” (i.e., those who take the ES course when ITT = 1 and do not when ITT = 0). For example, it may be that the ES course has more impact among those who only take it when encouraged than among the “always takers” who take the course regardless of their ITT status. We subset the sample by ES uptake and estimate auxiliary RD regressions (*SI Appendix*, Tables S9 and S10) for our high school persistence and educational-attainment measures. The key insight is that the contrast for ES enrollees (i.e., ES = 1) compares compliers and always takers (i.e., ITT = 1) with always takers (ITT = 0) above the assignment threshold. We find uniformly positive effects for measures of high school engagement and graduation. This suggests that the ES course was uniquely helpful for compliers, those students who enrolled in ES because their grade 8 GPA was below 2.0, whereas the ES course had no impact on those students who would have always enrolled in ES if it were offered. For those who do not enroll in ES (i.e., ES = 0), we compared never takers (i.e., ITT = 1) with compliers and never takers (i.e., ITT = 0). We find no effects for these students, which suggests that students with low grade 8 GPAs who requested their counselor switch them from ES to health or a college readiness elective had underlying characteristics (e.g., high motivation) that positioned them for better outcomes. These findings suggest that ES may be less effective for students who would decline (i.e., never takers) or insist (i.e., always takers) on enrolling in ES.

## Conclusion

This study presents evidence from a preregistered RD design, that participation in a grade 9 ES course significantly increased the probability of graduating from high school for academically lower-performing students. We also find complementary evidence that the course increased behavioral engagement in high school (i.e., enrollment, attendance, and earned credits) and may have increased college attendance. Our findings are consistent with a long-standing and influential body of evidence on the educational impact of ES courses and CRP, more generally, and provide credibly causal evidence consistent with the prior evidence of a positive association between ES participation and high school graduation (10).

Our results highlight that ES courses can provide students with an opportunity to engage with critical, anti-racist content in a rigorous college-preparatory course that promotes academic success. We also note that these results are consistent with conceptual frameworks from community psychology and experimental evidence on the educational benefits of targeted social-psychological interventions that support students’ sense of identity and belongingness in school environments. That is, ES courses resemble unusually intensive, multifaceted, and sustained social-psychological interventions, particularly for students from historically marginalized groups. For example, ES courses give students an opportunity to learn about, appreciate, and celebrate their origins, as well as foreground the contributions of marginalized communities to US history and social life (i.e., school belongingness and values affirmations). Furthermore, the explicit discussions of power, structural racism, stereotypes, and hegemony prime student awareness of the many structural social hurdles they face (i.e., stereotype forewarning and external attribution).

Targeting the ES course to ninth graders may be a critical design feature. Social-psychological interventions typically begin at the start of the academic year when students’ social identities in the stylized and evaluative context of classrooms may be at their most malleable. By engaging rising ninth graders as they entered their “make or break” year (58) in a new school environment, the ES course appears to have disrupted recursive cycles of poor attendance and academic disengagement that contribute to the risk of eventually dropping out.

Notably, we observe positive effects among different subgroups of students defined by their racial/ethnic and gender identities. However, two dimensions of heterogeneity in the educational impact of the ES course merit special note. The first involves the localness of estimates based on this RD design (i.e., an ITT based on a low grade 8 GPA). Because this research design leverages the targeting of the course to academically at-risk students, our inferences may not generalize to students who had higher grade 8 GPAs. Second, using a technique introduced by Bertanha and Imbens (56), we find evidence that the benefits of the ES course are lower among students who would always choose to take the course as well as among those who choose never to take the course when given an option.

Furthermore, the potentially considerable logistical challenges of fielding effective ES courses at scale and with a high degree of fidelity are also noteworthy. The ES course examined in this study reflected several years of curriculum development tailored to the local context as well as corresponding professional development of self-selected educators who had access to institutional support (e.g., release time for planning) and guidance from external experts. Mandating the wide-scale availability of ES through policy without thoughtful curricular development and teacher training may not reproduce the educational gains we document here. However, we also note that an ES course is likely to be exceptionally cost-effective relative to other interventions that reduce dropout risk. That is because the introduction of an ES course is likely to be a reallocation of existing resources dedicated to staffing, curricular development, and teacher training. In contrast, other interventions effective at promoting high school completion often require new and comparatively expensive out-of-class programming (e.g., cognitive behavioral therapy, ref. 45).

The results of this study comport with a research literature which suggests that the adoption of high-quality curricula is a relatively tractable lever for school reform (59). However, the unique features of the ES course make a distinctive contribution to this literature. Specifically, it suggests the educational potency of pedagogies and content (i.e., anti-racist education) that allow students to experience belongingness and psychological integrity in school. The results presented here suggest that the educational effects of curricula and teaching practices that similarly support positive social identities in classrooms merit further scrutiny in the context of other academic subjects (e.g., math, science, and reading).

## Supplementary Material

Supplementary File

## Data Availability

Data cannot be shared (data come from the administrative records of a school district including children under the age of 18. Per our data usage agreement with the school district, we are not able to share these data with others who have not been approved to access these data by the school district's internal research review process. Researchers must seek independent approval to access these data).

## Footnotes

*Our study preregistration is available here: http://osf.io/89mrt. Prior to having access to the data, we identified high school graduation as our confirmatory outcome.

^†^Students could opt out of the ES course and choose another elective by speaking with a counselor. We note that, following Dee and Penner (11), this sample excludes students with missing or outlier values for grade 9 GPA (i.e., a perfect 4.0 or a value less than 1.25), the assignment variable in this RD design.

^‡^While our study sample is distinct, we find no differences in student baseline test scores, racial/ethnic identification, sex, grade 8 attendance, or participation in English language or special education programming at the 2.0 GPA assignment threshold for encouragement in ES enrollment (*SI Appendix*, Table S8).

^§^Most of this missingness is due to the statewide data on high school graduates. Using the NSC enrollment data, we could confidently identify only three additional students as high school graduates. These students enrolled seamlessly (i.e., the summer or fall after their expected high school graduation date) in a 4-y institution. We chose not to categorize students who enrolled in 2-y colleges as high school graduates because students in our sample often enrolled in California Community Colleges, which do not require a high school diploma for individuals aged 18 and older. All students in our sample meet the age threshold, and therefore 2-y postsecondary enrollment is unreliable for imputing high school graduation.

^¶^Interestingly, the patterns in this missingness foreshadow this study’s main finding. Auxiliary RD regressions indicate that students with grade 8 GPAs below 2.0 (i.e., ITT = 1) are more likely than students just above the threshold (ITT = 0) to appear in the statewide records on high school graduates.

^#^We also present additional evidence consistent with the “exclusion restriction” (i.e., that the contrast at this threshold does not imply outcome-relevant determinants other than ES take-up). In *SI Appendix*, Tables S6 and S7, we find that this threshold has small and statistically insignificant effects on outcomes among students at the SFUSD high schools which did not offer the ES course.

^||^Unrestrictive histograms are consistent with this assumption as well but also indicate that values of the grade 8 GPA clearly cluster into “heaps” at integer and half-integer values. To investigate the possibility of “heaping-induced bias,” Dee and Penner (11) examine their results excluding observations with values of the assignment variable at these heaps (51). We conduct a similar exercise and find similar RD estimates in specifications that exclude these observations.

**Our main specifications are unchanged when we add these test score controls to our main results (*SI Appendix*, Table S3).

^††^In *SI Appendix*, Tables S4 and S5, we provide estimates of the “placebo” effect of EWI identification at schools that did not implement ES courses and find null impacts for high school engagement (*SI Appendix*, Table S4) and educational attainment (*SI Appendix*, Table S5).

^‡‡^We also estimate these results using different measures of postsecondary enrollment (i.e., 2-y only, 4-y only) and do not find substantially different results. We can view the specific institutions students near the ITT threshold enrolled in, and the increases in postsecondary enrollment are driven by broad-access institutions (e.g., California State Universities and community colleges).

^§§^We include the results from algorithmically chosen bandwidths (52, 53). We also note that the estimated impact on high school graduation becomes statistically insignificant in only two instances: when conditioning on a quadratic of the assignment variable and when using the data (i.e., 126 out of 1,405 observations) in the bandwidth based on the Calonico et al. (53) procedure. In both cases, the point estimate is largely unchanged, but there is a substantial loss of statistical precision.
