# Supplementary material for: Ethnic studies increases longer-run academic engagement and attainment
Source: Proc Natl Acad Sci U S A. 2021 Sep 7;118(37):e2026386118. doi: 10.1073/pnas.2026386118 (PMC8449416; doi:10.1073/pnas.2026386118)
Supplement: Supplementary File [file pnas.2026386118.sapp.pdf]

Appendix Table 1: Reduced-form RD estimates of educational attainment

Dependent variable	Full sample		Bandwidth |Grade-8 GPA_i| ≤ 0.67		mean
5-year state-confirmed graduate,	0.188**	0.155*	0.331***	0.294***	0.900
unknown imputed as non-graduates	(0.086)	(0.082)	(0.101)	(0.099)	
5-year state-confirmed graduate,	0.174**	0.140*	0.311***	0.273***	0.902
unknown non-graduates + NSC imputation	(0.086)	(0.082)	(0.101)	(0.098)	
5-year state-confirmed graduate,	0.185**	0.157**	0.279***	0.251***	0.911
NSC + on-track graduate imputed	(0.073)	(0.073)	(0.088)	(0.087)	
5-year state-confirmed graduate,	0.190***	0.161**	0.251***	0.244***	0.923
NSC + moderate off-track graduate imputed	(0.072)	(0.071)	(0.085)	(0.086)	
Postsecondary enrollment, year 5	0.163	0.134	0.289**	0.260**	0.695
	(0.101)	(0.0920)	(0.115)	(0.105)	
Postsecondary enrollment, year 6	0.128	0.083	0.284**	0.249**	0.681
	(0.100)	(0.095)	(0.113)	(0.104)	
Linear Splines	Yes	Yes	Yes	Yes	
Full controls	-	Yes	-	Yes	

Notes: Each cell contains the result of a separate regression of the effect of I(Grade-8 $GPA_i < 2.0$) on educational attainment measures. 5.6 percent of the sample have an unknown graduation outcome (i.e., left the district and did not enroll in a public school in California). We impute graduation outcomes for these students first by counting all leavers as graduates (top row); we impute all leavers to be non-graduates (2nd row); impute as graduates those with seamless postsecondary enrollment (3rd row); impute as graduates those who met internal district "on-track" benchmarks for credits earned at the time they left the district, our preferred measure, (4th row); impute as graduates those who were moderately (up to 1 year) "off-track" for credits earned at the time they left the district (5th row). Demographic controls include student sex and race/ethnicity. The full controls include grade-8 (i.e., prior to treatment status) special education identification, English learner status, attendance and suspension history. Grade-8 GPA is centered at 2.0. Robust standard errors are reported in parentheses. * p < .10 ** p < .05 *** p < .01

Appendix Table 2: Auxiliary RD estimates of baseline covariate balance

Dependent variable	Full sample		n	mean
Available Grade-8 ELA Score	0.00140	0.0813	1405	0.908
	(0.063)	(0.093)		
Available Grade-8 History Score	0.00907	0.00968	1405	0.987
	(0.007)	(0.012)		
Available Grade-8 Math Score	-0.0277	0.0195	1405	0.932
	(0.058)	(0.088)		
Grade-8 ELA Score, standardized	-0.0318	-0.104	1276	-0.065
	(0.287)	(0.224)		
Grade-8 History Score, standardized	0.230	0.149	1387	-0.086
	(0.241)	(0.216)		
Grade-8 Math Score, standardized	0.201	0.0159	1310	0.0658
	(0.247)	(0.239)		
Female	0.0142	-0.156	1405	0.417
	(0.090)	(0.148)		
Black	0.0899	-0.0333	1405	0.063
	(0.071)	(0.113)		
Hispanic	-0.0401	-0.0984	1405	0.868
	(0.098)	(0.176)		
Asian	0.0643	0.205	1405	0.601
	(0.098)	(0.175)		
Grade-8, Special-education	-0.00924	-0.0506	1405	0.123
	(0.078)	(0.120)		
Grade-8, English-learner	-0.0415	0.0217	1405	0.184
	(0.092)	(0.176)		
Grade-8, Ever suspended	0.00897	-0.0910*	1405	0.018
	(0.036)	(0.044)		
Grade-8, Attendance	1.198	2.073	1405	96.684
	(0.658)	(1.112)		
Linear Splines	Yes	Yes		
Quadratic Splines	-	Yes		

Notes: Each cell contains the result of a separate regression of I(Grade-8 $GPA_i < 2.0$) on baseline covariates. Test scores are standardized using the full sample of school district grade-8 students who participated in the exam. Grade-8 GPA is centered at 2.0. Robust standard errors are reported in parentheses.

* p < .05 ** p < .01 *** p < .001

Appendix Table 3. Auxilliary reduced form estimates of effect on educational attainment, by sample

Sample	Dependent Variable: High-school Graduation			Proportion of Full Sample
Full Sample	0.185**	0.157**	--	1.000
	(0.073)	(0.073)		
Grade-8 History	0.180*	0.155*	0.146*	0.987
	(0.073)	(0.072)	(0.072)	
Grade-8 Math	0.195*	0.166*	0.159*	0.932
	(0.077)	(0.075)	(0.075)	
Grade-8 ELA	0.188*	0.162*	0.157*	0.908
	(0.077)	(0.075)	(0.075)	
Grade-8 Math, History, & ELA	0.198**	0.172*	0.161*	0.900
	(0.076)	(0.074)	(0.075)	
Linear Splines	Yes	Yes	Yes	
Full controls	-	Yes	Yes	
Baseline test scores	-	-	Yes	

Notes: Each cell contains the result of a separate regression of I(Grade-8 $GPA_i < 2.0$) on high school graduation for distinct samples. Demographic controls include student sex and race/ethnicity. The full controls include grade 8 (i.e., prior to treatment status) special-education identification, English-learner status, attendance and suspension history. The third column includes baseline test score controls according to test score availability (see Sample column). Grade-8 GPA is centered at 2.0. Robust standard errors are reported in parentheses. * $p < .10$ ** $p < .05$ *** $p < .01$

Appendix Table 4. Reduced-form placebo RD effects on high school persistence, by year

	(1)	(2)	(3)	(4)	(5)	(6)
Dependent variable	Full Sample			Bandwidth $\lvert$Grade-8 $GPA_i\rvert \leq 0.67$		
	estimate	n	mean	estimate	n	mean
Enrolled in district, year 2	0.0496<br>(0.0322)	2861	0.973	0.0554<br>(0.040)	655	0.955
Attendance, year 2	-3.395<br>(2.935)	2783	94.402	-2.406<br>(3.346)	615	88.107
Credits earned, year 2	-5.924<br>(5.438)	2783	120.785	-2.003<br>(6.506)	615	83.566
Enrolled in district, year 3	-0.00161<br>(0.0561)	2861	0.949	-0.00242<br>(0.064)	655	0.911
Attendance, year 3	-5.661<br>(3.627)	2715	93.111	-5.038<br>(4.380)	587	85.582
Credits earned, year 3	-5.716<br>(7.731)	2715	182.445	-0.237<br>(9.405)	587	159.797
Enrolled in district, year 4	0.0239<br>(0.0627)	2861	0.932	0.0126<br>(0.074)	655	0.873
Attendance, year 4	0.540<br>(3.468)	2667	90.955	3.283<br>(4.218)	562	83.566
Credits earned, year 4	-5.367<br>(8.502)	2667	243.768	1.801<br>(10.815)	562	222.350

Notes: Each cell contains the result of a separate regression of the placebo (i.e., early warning indicator (EWI) label only) effect of I(Grade-8 $GPA_i < 2.0$) on high-school persistence measures (i.e., district enrollment, attendance and credits earned) from administrative data. The placebo sample includes students at district schools that did not implement the Ethnic Studies curriculum. All models include linear splines and a full set of demographic controls (i.e., student sex and race/ethnicity indicators) and grade-8 (i.e., prior to treatment status) special-education identification, English-learner status, attendance and suspension history. Grade-8 GPA is centered at 2.0. Robust standard errors are reported in parentheses. * $p < .10$ ** $p < .05$ *** $p < .01$

Appendix Table 5. Reduced-form placebo RD estimates of educational attainment

Dependent variable	Full sample		Bandwidth |Grade-8 GPA_i| ≤ 0.67		mean
5-year state-confirmed graduate, unknown imputed as non-graduates	0.0161	0.00716	0.0183	0.0197	0.886
	(0.073)	(0.073)	(0.086)	(0.088)	
5-year state-confirmed graduate, unknown non-graduates + NSC imputation	0.0161	0.00716	0.0219	0.0265	0.886
	(0.073)	(0.073)	(0.087)	(0.089)	
5-year state-confirmed graduate, NSC + on-track graduate imputed	0.0305	0.0232	0.0591	0.0626	0.908
	(0.069)	(0.068)	(0.083)	(0.083)	
5-year state-confirmed graduate, NSC + moderate off-track graduate imputed	-0.0208	-0.0282	(0.080)	0.0197	0.921
	(0.067)	(0.067)	(0.080)	(0.080)	
Postsecondary enrollment, year 5	-0.0588	-0.0682	-0.0632	-0.0746	0.714
	(0.0773)	(0.0763)	(0.094)	(0.094)	
Postsecondary enrollment, year 6	-0.0725	-0.0810	-0.0852	-0.0887	0.678
	(0.075)	(0.073)	(0.092)	(0.090)	
Linear Splines	Yes	Yes	Yes	Yes	
Full controls	-	Yes	-	Yes	

Notes: Each cell contains the result of a separate regression of the placebo effect (i.e., Early Warning Indicator (EWI) label only) of I(Grade-8 $GPA_i < 2.0$) on educational attainment measures. The placebo sample includes students at district schools that did not implement the Ethnic Studies curriculum. 8.4 percent of the sample have an unknown graduation outcome (i.e., left the district and did not enroll in a public school in California). We impute graduation outcomes for these students first by counting all leavers as graduates (top row); we impute all leavers to be non-graduates (2nd row); impute as graduates those with seamless postsecondary enrollment (3rd row); impute as graduates those who met internal district "on-track" benchmarks for credits earned at the time they left the district, our preferred measure, (4th row); impute as graduates those who were moderately (up to 1 year) "off-track" for credits earned at the time they left the district (5th row). Demographic controls include student sex and race/ethnicity. The full controls include grade-8 (i.e., prior to treatment status) special-education identification, English-learner status, attendance and suspension history. Grade-8 GPA is centered at 2.0. Robust standard errors are reported in parentheses. * p < .10 ** p < .05 *** p < .01

Appendix Table 6. Reduced-form Difference in Discontinuity effects on outcomes

	(1)	(2)	(3)	(4)
Dependent variable	RD Estimates		Difference in Discontinuity Estimates	
	estimate	n	estimate	n
High-school graduate	0.157**<br>(0.073)	1405	0.151*<br>(0.073)	4266
Enrolled in district, year 2	-0.00987<br>(0.0419)	1405	-0.0535<br>(0.0351)	4266
Attendance, year 2	5.741*<br>(3.065)	1369	11.07***<br>(3.130)	4152
Credits earned, year 2	8.014*<br>(4.794)	1369	13.83**<br>(5.948)	4152
Enrolled in district, year 3	0.00203<br>(0.0516)	1405	-0.00381<br>(0.0746)	4266
Attendance, year 3	6.323**<br>(2.989)	1333	11.37***<br>(3.781)	4048
Credits earned, year 3	10.56<br>(6.570)	1333	16.04*<br>(9.224)	4048
Enrolled in district, year 4	0.0374<br>(0.0606)	1405	0.0262<br>(0.0856)	4266
Attendance, year 4	7.158***<br>(1.799)	1304	5.626<br>(4.083)	3971
Credits earned, year 4	15.29**<br>(7.724)	1304	20.16*<br>(10.07)	3971

Notes: Each cell contains the result of a separate regression of the effect of I(Grade-8 $GPA_i < 2.0$) interacted with an indicator for attending an ES pilot school (i.e., ITT=1) on high-school graduation and persistence measures (i.e., district enrollment, attendance and credits earned) from administrative data. These models estimate the reduced form effect of ES eligibility conditioned on a placebo jump for those students at EWI-only intervention schools (i.e., difference in discontinuity). As such, all models include a linear spline of the forcing variable (i.e., Grade-8 GPA), an indicator for Grade-8 GPA < 2.0, and those RD specification variables interacted with an indicator for attending an ES pilot school. Additionally, all models include school-year fixed effects (i.e., cohort fixed effects) and a full set of demographic controls (i.e., student sex and race/ethnicity indicators) and grade-8 (i.e., prior to treatment status) special-education identification, English-learner status, attendance and suspension history. Grade-8 GPA is centered at 2.0. Robust standard errors are reported in parentheses. * p < .10 ** p < .05 *** p < .01

Appendix Table 7. Reduced-form RD estimates of high-school persistence by student traits

	(1)	(2)	(3)		(4)	(5)	(6)	(7)	(8)	(9)	(10)
	Enrolled in district,			Sample	Attendance,			Credits earned,			Sample Size
	year 2	year 3	year 4	Size	year 2	year 3	year 4	year 2	year 3	year 4	
**Sample**											
Full sample	-0.00754	-0.00843	0.0479	1405	7.552***	5.741*	6.323**	8.014*	10.56	15.29**	1369 / 1333 / 1304
	(0.042)	(0.051)	(0.061)		(1.840)	(3.065)	(2.989)	(4.794)	(6.570)	(7.724)	
Male	-0.0269	-0.0253	0.0428	819	8.072***	3.414	5.368	8.887	5.269	9.547	798 / 775 / 760
	(0.053)	(0.064)	(0.077)		(2.253)	(3.737)	(3.749)	(5.571)	(7.524)	(9.210)	
Female	0.0725*	0.0954*	0.121	586	5.458*	12.23***	7.961*	7.841	25.59**	34.28***	571 / 558 / 544
	(0.039)	(0.049)	(0.076)		(3.134)	(4.404)	(4.335)	(9.362)	(11.532)	(12.850)	
Hispanic	0.0286	-0.00686	0.0292	324	7.541**	0.505	7.151	10.37	21.83*	25.37*	312 / 306 / 299
	(0.052)	(0.061)	(0.094)		(3.341)	(5.957)	(5.945)	(8.200)	(12.292)	(14.274)	
Asian	-0.0433	-0.000495	0.121	845	4.301**	7.081**	5.185**	5.366	5.395	4.149	832 / 818 / 807
	(0.082)	(0.085)	(0.101)		(1.804)	(3.328)	(2.433)	(7.238)	(7.238)	(9.023)	

Notes: Each cell contains the results of a separate regression of the effect of I(Grade-8 $GPA_i < 2.0$) on measures of high-school persistence (i.e., enrollment, attendance and credits earned). Demographic controls include student sex and race/ethnicity. All models include linear splines, cohort-by-school fixed effects, and controls for student sex and race/ethnicity and grade-8 (i.e., prior to treatment status) special-education identification, English-learner status, attendance and suspension history. Grade-8 GPA is centered at 2.0. Robust standard errors are reported in parentheses. * p < .10 ** p < .05 *** p < .01

Appendix Table 8. Reduced-form RD estimates of educational attainment by student traits

	(1)	(2)	(3)	(4)	(5)	(6)	(7)
	High-school Graduate, year 5		Postsecondary enrollment, year 5		Postsecondary enrollment, year 6		Sample Size
**Sample**							
Full sample	0.157**	0.251***	0.134	0.260**	0.149	0.249**	1405 / 424
	(0.073)	(0.087)	(0.092)	(0.105)	(0.091)	(0.104)	
Male	0.150*	0.240**	0.0881	0.208*	0.145	0.247**	819 / 301
	(0.087)	(0.102)	(0.106)	(0.124)	(0.106)	(0.122)	
Female	0.233*	0.368**	0.333*	0.381*	0.204	0.191	586 / 123
	(0.120)	(0.152)	(0.188)	(0.220)	(0.200)	(0.233)	
Hispanic	0.112	0.181	-0.0389	0.0918	-0.00807	0.146	324 / 158
	(0.133)	(0.154)	(0.150)	(0.174)	(0.146)	(0.163)	
Asian	0.187*	0.244*	0.248*	0.368**	0.204	0.319*	845 / 165
	(0.103)	(0.125)	(0.133)	(0.165)	(0.143)	(0.176)	
Bandwidth	Full	1SD	Full	1SD	Full	1SD	Full / 1SD

Notes: Each cell contains the results of a separate regression of the effect of I(Grade-8 $GPA_i < 2.0$) on educational attainment (i.e., high-school graduation and postsecondary enrollment). High-school graduation is our preferred measure that includes imputed outcomes for district and state leavers (i.e., enrolled in private school, public school out-of-state or left country) who enrolled seamlessly in postsecondary or were on-track to graduate based on internal district benchmarks for credits earned. All models include linear splines, cohort-by-school fixed effects, and controls for student sex and race/ethnicity and grade-8 (i.e., prior to treatment status) special-education identification, English-learner status, attendance and suspension history. Grade-8 GPA is centered at 2.0. Robust standard errors are reported in parentheses. * $p < .10$ ** $p < .05$ *** $p < .01$

Appendix Table 9. Reduced-form RD estimates of high-school persistence by complier status

	(1)	(2)	(3)		(4)	(5)	(6)	(7)	(8)	(9)	(10)
	Enrolled in district,			Sample	Attendance,			Credits earned,			Sample Size
	year 2	year 3	year 4	Size	year 2	year 3	year 4	year 2	year 3	year 4	
**Sample**											
Full sample	-0.00754	-0.00843	0.0479	1405	7.552***	5.741*	6.323**	8.014*	10.56	15.29**	1369 / 1333 / 1304
	(0.042)	(0.051)	(0.061)		(1.840)	(3.065)	(2.989)	(4.794)	(6.570)	(7.724)	
ES= 1	-0.000238	-0.0303	0.0834	179	7.684***	8.689**	8.028	12.74*	15.59	17.34	170 / 167 / 159
	(0.057)	(0.070)	(0.093)		(2.388)	(4.034)	(6.142)	(7.245)	(10.179)	(13.806)	
ES= 0	-0.0629	-0.0393	-0.0152	1226	4.214	1.065	6.009*	-1.605	2.567	13.94	1199 / 1166 / 1145
	(0.067)	(0.083)	(0.099)		(2.894)	(5.026)	(3.620)	(7.098)	(10.009)	(10.350)	

Notes: Each cell contains the results of a separate regression of the effect of I(Grade-8 $GPA_i < 2.0$) on measures of high-school persistence (i.e., enrollment, attendance and credits earned). Demographic controls include student sex and race/ethnicity. All models include linear splines, cohort-by-school fixed effects, and controls for student sex and race/ethnicity and grade-8 (i.e., prior to treatment status) special-education identification, English-learner status, attendance and suspension history. Grade-8 GPA is centered at 2.0. Robust standard errors are reported in parentheses. * p < .10 ** p < .05 *** p < .01

To implement to Bertanha & Imbens (2020) procedure, we examine our reduced-form RD results in separate sub-samples of the data where ES=1 and where ES=0. When ES=1, this RD estimate separates students to the right of the threshold (i.e., ITT=0) who are exclusively "always takers" from the population to the left of the threshold (ITT=1) which consists of both "always takers" and "compliers." In this case, a statistically significant discontinuity in outcomes at the threshold would be consistent with the hypothesis of treatment heterogeneity across these groups. Similarly, in the sub-sample of data where ES=0, the threshold separates "never takers" (i.e., ITT=1) from a population of "never takers" and "compliers" to the right of the threshold (i.e., ITT=0). A discontinuity in outcomes among students in this sample would suggest heterogeneity in the outcome-relevant but unobserved determinants of student success across these populations.

Appendix Table 10. Reduced-form RD estimates of educational attainment by complier status

	(1)	(2)	(3)	(4)	(5)	(6)	(7)
	High-school Graduate, year 5		Postsecondary enrollment, year 5		Postsecondary enrollment, year 6		Sample Size
**Sample**							
Full sample	0.157**	0.251***	0.134	0.260**	0.149	0.249**	1405 / 424
	(0.073)	(0.087)	(0.092)	(0.105)	(0.091)	(0.104)	
ES = 1	0.340***	0.433**	0.150	0.392*	0.131	0.286	179 / 113
	(0.104)	(0.175)	(0.160)	(0.224)	(0.162)	(0.220)	
ES = 0	0.0363	0.105	0.107	0.229	0.148	0.253*	1226 / 311
	(0.119)	(0.132)	(0.127)	(0.143)	(0.123)	(0.142)	
Bandwidth	Full	1SD	Full	1SD	Full	1SD	Full / 1SD

Notes: Each cell contains the results of a separate regression of the effect of I(Grade-8 $GPA_i < 2.0$) on educational attainment (i.e., high-school graduation and postsecondary enrollment). High-school graduation is our preferred measure that includes imputed outcomes for district and state leavers (i.e., enrolled in private school, public school out-of-state or left country) who enrolled seamlessly in postsecondary or were on-track to graduate based on internal district benchmarks for credits earned. All models include linear splines, cohort-by-school fixed effects, and controls for student sex and race/ethnicity and grade-8 (i.e., prior to treatment status) special-education identification, English-learner status, attendance and suspension history. Grade-8 GPA is centered at 2.0. Robust standard errors are reported in parentheses.
* p < .10 ** p < .05 *** p < .01
